# Improvement of Gait Dysfunction after Applying a Hinged Ankle–Foot Orthosis in a Hemiplegic Cerebral Palsy Patient with Disrupted Medial Lemniscus: A Case Report

**DOI:** 10.3390/children8020081

**Published:** 2021-01-25

**Authors:** Su Min Son, Min Cheol Chang

**Affiliations:** Department of Physical Medicine & Rehabilitation, College of Medicine, Yeungnam University, Daegu 712837, Korea; sumin430@hanmail.net

**Keywords:** cerebral palsy, proprioception, medical lemniscus, diffusion tensor tractography, gait, ankle-foot orthosis

## Abstract

We describe the successful application of hinged ankle−foot orthoses (AFOs) in a cerebral palsied (CP) patient with gait instability due to a disrupted medial lemniscus (ML). The patient was a 27-month-old male CP child with gait instability who presented with reduced knee flexion and ankle dorsiflexion, with severe genu recurvatum on his right lower extremity during gait. The patient had no motor weakness or spasticity. Conventional magnetic resonance imaging (MRI) revealed no definite abnormal lesion. However, diffusion tensor tractography (DTT) showed disruption of the left ML, consistent with right hemiplegic symptoms. The integrity of the major motor-related neural tracts, including the corticospinal and corticoreticulospinal tracts, was preserved. We considered that the patient’s abnormal gait pattern was related to the disrupted ML state. We applied hinged AFOs, which immediately resulted in a significantly stabilized gait. The angles of knee flexion and ankle dorsiflexion increased. Our findings indicate that the application of hinged AFOs could be a useful therapeutic option for CP patients with gait instability related to ML disruption. In addition, we showed that DTT is a useful tool for identifying the causative brain pathology in CP patients, especially when conventional brain MRIs show no specific lesion.

## 1. Introduction

Cerebral palsy (CP) is a term used to define a broad range of disorders of movement, posture, or tone, resulting from a non-progressive lesion in the immature brain [1]. CP patients are known to have various neural tract disorders [2] and present with various symptoms depending on which neural tract is involved. However, many of these patients show no definite abnormal lesion in conventional brain magnetic resonance imaging (MRI) [3].

The medial lemniscus (ML) is one of the main somatosensory pathways in the human brain. It conveys proprioceptive information, which is conscious awareness of the body position in space [4]. ML deterioration causes postural imbalance and gait disturbance, which is known to be a cause of CP [5].

The application of specific treatment strategies for the exact diagnosis and specific symptoms in CP patients is crucial to obtain better outcomes. Evaluation of the neural tract is helpful for appropriate personalized treatment in CP patients. Diffusion tensor tractography (DTT) is a powerful noninvasive imaging technique that can identify and represent the neural tracts in the human brain [5,6,7] and can be applied to investigate the state of neural tracts in CP patients [3,5,6].

In this study, we aimed to demonstrate abnormal hemiplegic gait instability in our patient, related to the disruption of the corresponding ML, and report on the successful application of hinged ankle–foot orthoses (AFOs) for improving gait stability.

## 2. Case Presentation

A 27-month-old male CP patient was brought to the physical medicine and rehabilitation department of a university hospital for gait dysfunction. The patient was born at 30 weeks of gestation, had a birth weight of 3.52 kg, and had no specific perinatal history. He was able to walk independently at 23 months. His parents reported that he showed left-hand dominance before 12 months and mild torticollis to the right side, but no evaluation or treatment was performed for those symptoms. He could walk independently, but his gait was unstable and he would often fall. During gait, decreased knee flexion and ankle dorsiflexion with severe genu recurvatum were noted in his right lower limb. Physical examination revealed the absence of motor weakness and spasticity. Deep tendon reflexes (biceps, triceps, knee, and ankle jerks) were normal, and no pathologic reflexes were observed. Other tests, including tests for proprioception, could not be performed on the patient due to poor cooperation. Electromyography testing of the peripheral nervous system could not be performed due to the patient’s parents’ refusal. No abnormal findings were noted in conventional brain and spinal MRI.

To evaluate the state of the neural tracts, we performed diffusion tensor imaging (DTI). DTI data were obtained using a synergy-L Sensitivity Encoding 6-channel head coil on the 1.5T Philips Gyroscan Intera system (Hoffmann-La Roche, Best, The Netherlands). Sixty-seven contiguous slices were acquired parallel to the anterior commissure-posterior commissure line. The following imaging parameters were applied: matrix = 128 × 128, field of view = 221 mm × 221 mm, echo time = 76 ms, repetition time = 10,726 ms, parallel imaging reduction factor = 2, EPI factor = 67 and b = 1000 s/mm^2^, NEX = 1, and thickness = 2.3 mm (acquired voxel size = 1.73 mm × 1.73 mm × 2.3 mm).

**Figure 1:**
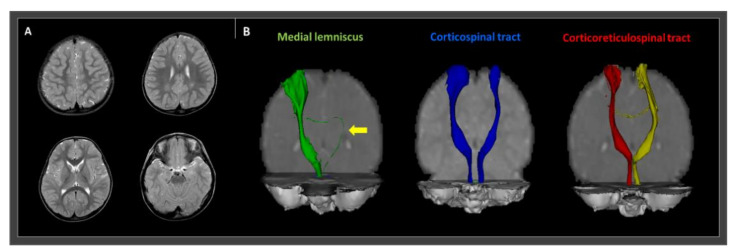
T2-weighted magnetic resonance imaging and diffusion tensor tractography (DTT) images of a 27-month old male cerebral palsied patient with gait instability. (**A**) T2-weighted magnetic resonance images show no abnormal lesions. (**B**) DTT revealing a thinned and disrupted left medial lemniscus (yellow arrow). However, the right medial lemniscus and both the corticospinal and corticoreticulospinal tract show preserved integrity to the cortex.

The Oxford Center for Functional Magnetic Resonance Image of the Brain (FMRIB) Software Library (FSL; http://www.fmrib.ox.ac.uk/fsl) was used to analyze the diffusion-weighted imaging data. Fiber tracking was performed using a probabilistic tractography method based on a multi-fiber model, applied using tractography routines implemented in FMRIB diffusion (5000 streamline samples, 0.5-mm step lengths, curvature thresholds = 0.2). The ML, corticospinal tract (CST), and corticoreticulospinal tract (CRT) were depicted by selecting fibers passing through both regions of interest (ROIs) [7,8]. For ML integrity analysis, the seed ROI was placed on the medial posterior region of the medullary pyramids and the target ROI was placed on the ventroposterolateral nucleus of the thalamus. A seed ROI of the CST was placed on the CST portion of the anterior mid-pons on a two-dimensional (2D) color map. For CST, the target ROI was located on the CST portion of the anterior lower pons, again on a 2D color map. To analyze the state of the CRT, a seed ROI was positioned on the reticular formation in the medulla. The first target ROI was positioned on the midbrain tegmentum, and the second was positioned on the primary motor cortex, specifically in Brodmann area 6. Fiber tracking of these three neural tracts was initiated at the center of the seed voxel with a fractional anisotropy >0.2 and ended at the voxel having a fiber assignment <0.2 and a tract-turning angle <60°. The integrity of the right ML was well-preserved, but the left ML was disrupted and significantly thinned. On the other hand, both the CST and CRT were well depicted without any significant disruption (Figure 1).

**Figure 2:**
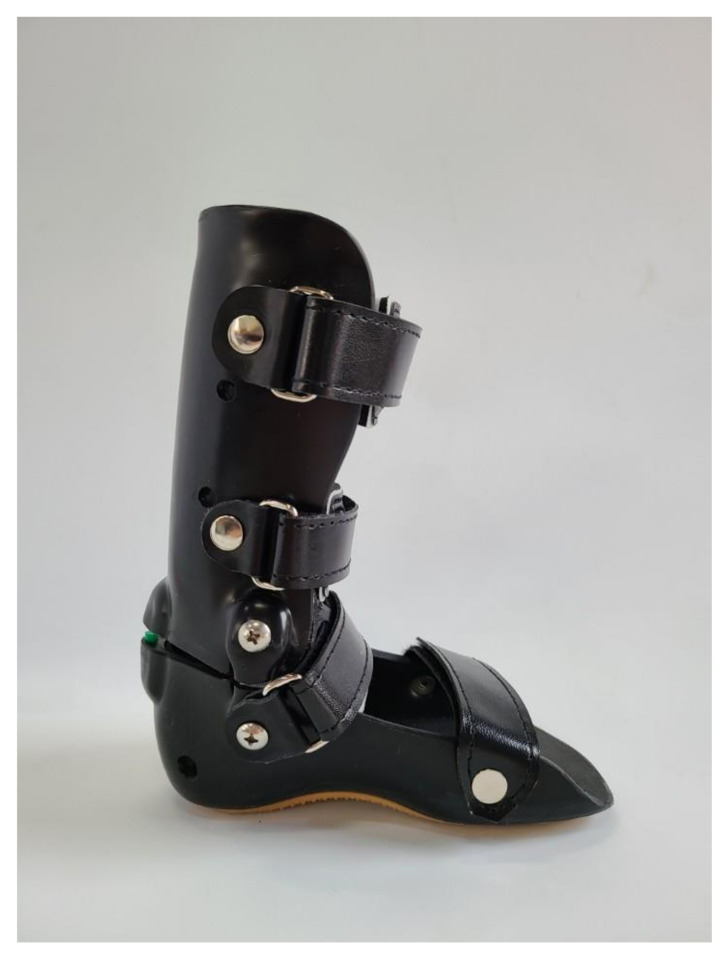
The hinged ankle–foot orthosis.

To increase knee flexion and ankle dorsiflexion and improve gait stability, we applied bilateral hinged AFOs (full-length footplates) (Figure 2). Although the patient’s gait abnormality showed a remarkable hemiplegic pattern on his right side, both AFOs were applied to increase overall gait stability. Immediately after applying AFOs, his gait significantly stabilized (Supplementary Video S1). Angles of knee flexion and ankle dorsiflexion increased. Severe genu recurvatum improved and approached a normal gait pattern.

## 3. Discussion

In this study, we reported successful application of hinged AFOs for the treatment of gait dysfunction in a patient with CP related to ML. Conventional MRI revealed no specific lesion, and DTT showed intact major motor-related neural tracts, including CST and CRT. However, the integrity of the left ML was disrupted, and this result was consistent with the patient’s right hemiplegic pattern. The ML is a somatosensory-related neural tract that transfers information on proprioception, fine touch, and vibratory information (body position and kinesthetic sense) [4]. Therefore, a lesion of the ML can result in gait dysfunction. In our patient, reduced angles of knee flexion and ankle dorsiflexion on his right lower limb during gait would have been a compensatory phenomenon for impaired gait stability related to disruption of the left ML. The development of CP by impairment of the ML was reported by Jung et al. [5]. They reported 13 hemiplegic patients having CP without motor weakness and spasticity, whose hemiplegic symptoms were related to the corresponding affected ML. In the present case, the patient’s parents reported his left-hand dominance before 12 months and mild torticollis. We believe that these symptoms might also be hemiplegic symptoms related to the state of the patient’s ML.

DTT is an imaging technique that can represent the neural tracts of the human brain in vivo [9,10,11]. It can depict various neural tracts using information on directionality of the movement of water molecules along the neural tracts of white matter [12]. It is being used in the evaluation of the state of the neural tracts in various brain disorders such as stroke, traumatic brain injury, and dementia [9,10,11]. In addition, DTT is helpful for the diagnosis of CP that shows no specific abnormality in conventional brain MRI [5,13]. Similar to previous studies, our patient showed no brain lesion in conventional MRI despite definite hemiplegic symptoms. We found a brain pathology corresponding to the hemiplegic abnormal gait pattern using DTT.

An AFO is an orthosis that surrounds the ankle and at least part of the foot and is applied to the external surface of the ankle to control its movement and position, as well as compensate for weakness of ankle dorsiflexion [14]. AFO has been reported to improve stabilization of the lower extremities and balance of standing and gait in older adults [14]. Likewise, after the application of AFOs in our patient, gait instability significantly improved. In addition, the hinge in the AFO plays a role in preventing ankle plantar flexion but allowing ankle dorsiflexion; therefore, it can increase the angle of ankle dorsiflexion during the swing phase of the gait cycle [15]. Because our patient showed a reduced angle of ankle dorsiflexion during gait, we applied a hinged AFO. The hinged AFOs could have improved ankle dorsiflexion, which also increased the angle of knee flexion and decreased genu recurvatum during gait. 

In conclusion, we reported on a hemiplegic CP patient whose gait instability was related to disruption of the corresponding ML, which significantly improved after the application of hinged AFOs. Thus, the application of hinged AFOs would be helpful for enhancing the gait stability of CP patients with ML disruption. This is the first study to show successful treatment of gait instability related to ML disruption using hinged AFOs in CP patients. In addition, we showed that DTT is useful for determining the causative brain pathology in CP patients, especially when conventional brain MRIs show no specific lesion. However, our study is limited in that it is a case report. Further studies involving large numbers of patients are warranted in the future. 

## Data Availability

All data are provided either in the paper or in Supplementary Materials.

## Supplementary Materials

The following are available online at https://www.mdpi.com/2227-9067/8/2/81/s1, Video S1. The patient’s gait before and after applying a hinged ankle-foot orthosis.
